# Loss of LRP1 in Adult Neural Stem Cells Impairs Migration to Ischemic Lesions

**DOI:** 10.1093/stmcls/sxad034

**Published:** 2023-04-25

**Authors:** Kristi Dietert, Swetha Mahesula, Sheetal Hegde, John Verschelde, Pamela Reed, Shane Sprague, Erzsebet Kokovay, Naomi L Sayre

**Affiliations:** University of Texas Health San Antonio, TX, USA; University of Texas Health San Antonio, TX, USA; University of Texas Health San Antonio, TX, USA; University of Texas Health San Antonio, TX, USA; University of Texas Health San Antonio, TX, USA; University of Texas Health San Antonio, TX, USA; University of Texas Health San Antonio, TX, USA; University of Texas Health San Antonio, TX, USA; South Texas Veteran’s Health Care System San Antonio, TX, USA

## Abstract

After ischemia, cells in the brain parenchyma upregulate stromal derived factor 1 (SDF1), driving chemokine receptor CXCR4-mediated migration of adult neural stem cells to the ischemic injury. We discovered a novel regulator of CXCR4 in neural stem cells, low-density lipoprotein receptor related protein 1 (LRP1). We used Nestin-driven knockout of LRP1 and induction of td-tomato in neural stem cells of adult mice. We observed reduced localization of td-tomato positive cells to the lesion, and find disrupted CXCR4-mediated neural stem cell migration in vitro, which is likely driven by LRP1-mediated loss of CXCR4 expression in vivo. Our results suggest that LRP1 is a novel regulator of CXCR4 in neural stem cells. This heretofore unknown interaction between LRP1 and CXCR4 could have significant consequences for multiple aspects of neural stem cell physiology.

## Graphical abstract

**Graphical Abstract:**
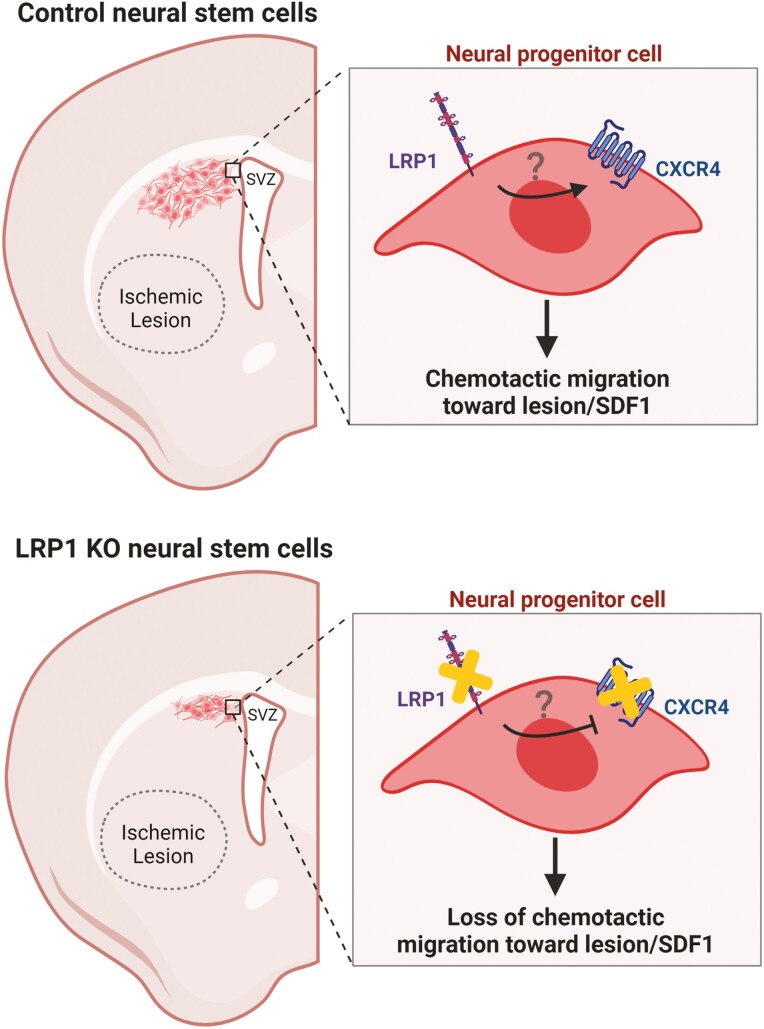

**Significance Statement**

CXCR4 plays an essential role in stem cells, particularly in regulating migration and proliferation. In adult neural stem cells, CXCR4 enables the migration of neural stem cells out of the subventricular niche toward ischemic lesions. After stroke, such migration could be harnessed to enhance the endogenous neuroprotective response. Here we show that LRP1 is a novel regulator of CXCR4 expression. Enhanced understanding of the relationship between CXCR4 and LRP1 would enable improved targeting not only of response to stroke but the function of CXCR4 in other contexts involving stem cell migration including cancer and hippocampal neurogenesis.

## Introduction

Stroke is a leading cause of death and a primary cause of disability worldwide. An estimated 87% of strokes are ischemic strokes, which arise from occlusion of blood flow.^1^ Currently, subacute therapeutics capable of mitigating post-stroke damage are lacking, necessitating improved understanding of mechanisms that drive post-stroke damage and repair.

Multiple signals are released in the ischemic lesion after stroke; neural stem cells (NSCs) resident within the subventricular zone (SVZ) niche are prime targets for such signals. After stroke, NSCs become activated and proliferate to expand the progenitor pool in the SVZ.^2^ The chemokine SDF1 (stromal cell-derived factor 1) is secreted by astrocytes and endothelial cells in the ischemic lesion, causing neuroblasts to divert from the rostral migratory stream (RMS) and instead migrate toward the lesion.^3,4^ Once there, they give rise to a limited number of neurons^5^ and secrete trophic factors.^6^ Ultimately, NSC migration toward the lesion is considered protective because genetic ablation of neural progenitors increases lesion sizes after stroke.^5-7^

The ability of NSCs to proliferate, migrate, and differentiate in a neuroprotective manner depends on dynamic and responsive cellular signaling. The low-density lipoprotein receptor related protein 1 (LRP1) is a key, albeit underappreciated, player in signal regulation. LRP1 is implicated in the binding/trafficking of over 40 proteins in multiple cell types. When bound to ligand/plasma membrane proteins, LRP1 undergoes receptor mediated endocytosis to traffic bound protein for degradation.^8^ LRP1 can also be cleaved by γ-secretase, translocating to the nucleus to regulate gene expression.^9^ Currently, the degree to which LRP1 influences signaling in NSCs is unclear, but many pathways relevant to NSC biology are regulated by LRP1.^8^

We sought to test a role for LRP1 in regulation of NSCs in the post-stroke milieu. We discovered that knockout of LRP1 in adult NSCs impaired migration toward ischemic lesions concomitant to a loss in CXCR4 expression. Altogether, our results suggest a heretofore undiscovered player in the regulation of CXCR4 in NSCs, which could have broad implications for NSC physiology in health and disease.

## Methods

### Animals

All animal procedures were approved by the UT Health Institutional Animal Care and Use Committee (IACUC) in accordance with NIH guidelines. Vivarium conditions included 12 h light/dark cycle with ad libitum food and water. Adult NSC-specific LRP1 KO mice were generated by crossing: Nestin-Cre^ERT2^ (Jackson stock #016261) to induce Cre recombinase in NSCs, tdTomato Ai14 reporter mice (tdTomato-stop^fl/fl^ stock 007914), and LRP1 floxed mice (stock 012604). KO was induced at 2 months of age with daily IP tamoxifen (100 μL of 20 mg/mL dissolved in corn oil) administered for 5 days. At 2 weeks post-stroke, mice were euthanized via 5% isoflurane/oxygen before being perfused with ice-cold PBS then 4% PFA/PBS.

### Transient MCAO

Using aseptic techniques, anesthetized mice (1%-3% isoflurane/oxygen) were maintained on a warming pad and an intraluminal filament was inserted into the left MCA via the carotid artery, similar to previously published procedures.^10^ A laser Doppler probe (Perimed) monitored blood flow. After 30 min of occlusion, the filament was removed. Sterile lactated ringers solution (1 mL SC daily-McKesson) and buprenorphine (0.05 mg/kg IP twice daily) was administered for 48 h post-surgery.

### Modified Neurological Severity Score

Neurological impairment was measured by a blinded female researcher using the modified neurological severity score (mNSS), similar to published procedures.^11^ Mice lacking deficits score a 0, while the most severe deficits score a 14.

### Statistical Analyses

All experiments and analyses were performed by a researcher who was blinded to the genotype/experimental manipulation. Relevant numbers of mice used for each experiment are noted as appropriate. Males and females were used in all conditions. For image analysis, at least 3-4 sections spaced every 150 µm were analyzed and pooled per mouse. For in vitro analysis, experiments were performed in triplicate independent experiments with *n* = 3+ wells/condition. Students *t*-test was used for 2-group comparisons and ANOVA with Tukey’s HSD was used to compare more than 2 groups with *α* set to 0.05. GraphPad Prism software was used for all statistical analyses. All results show the mean with standard error.

For methods involving cell culture, image analysis, immunohistochemistry, RNA extraction, and qRT-PCR, please see Supplementary information.

## Results

### Loss of LRP1 in Adult NSCs Impairs Localization to Ischemic Lesions

**Figure 1.**
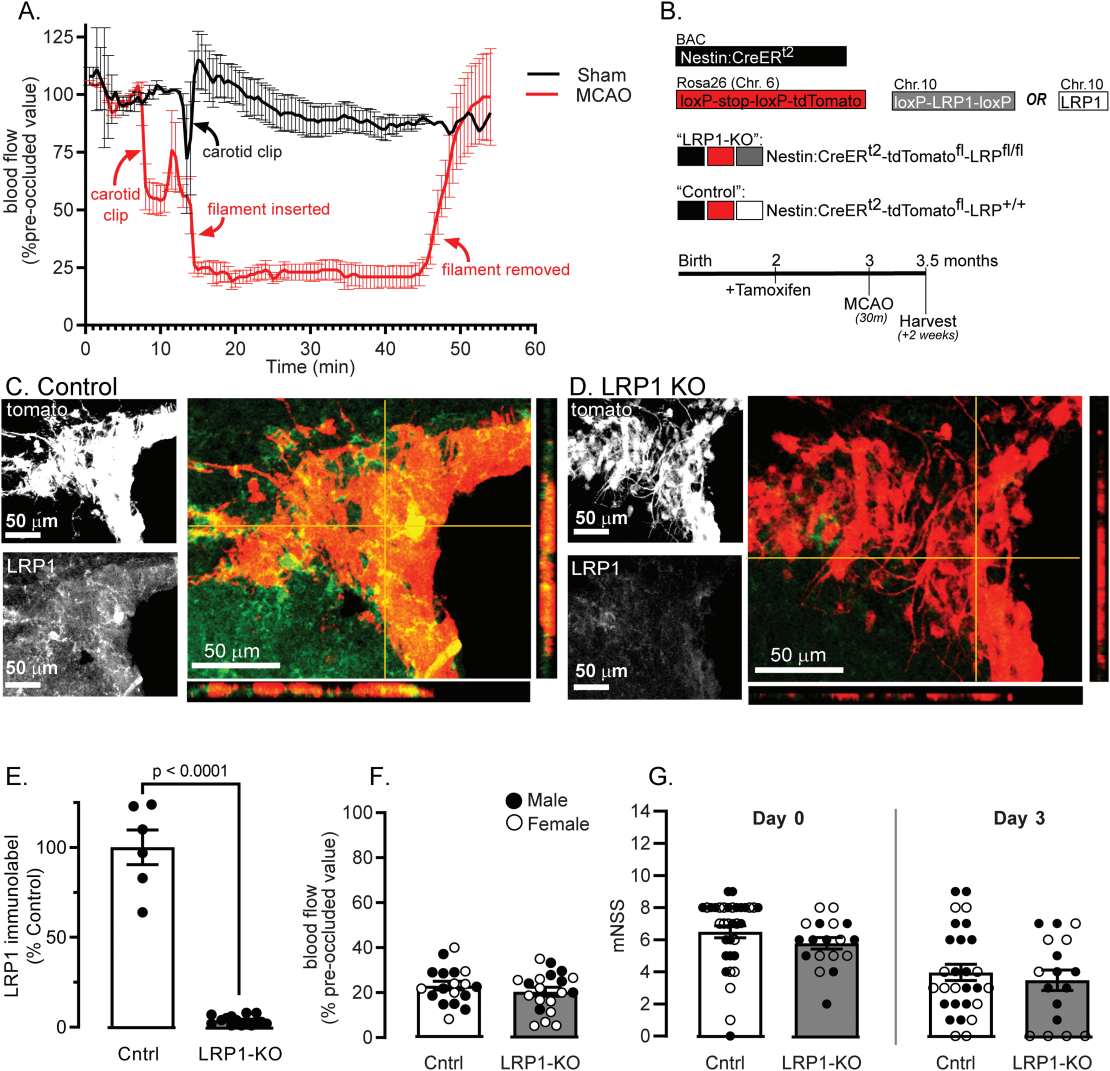
MCAO in inducible NSC-specific LRP1 KO mice. (**A**) Cortical perfusion was visualized via laser-Doppler in sham (black) and occluded (red) mice (*n* = 4/group). (**B**) Nestin:Cre^ERt2^:tdTomato mice were crossed with LRP1^fl/fl^ mice to create Nestin-Cre^ER^:td-tomato:LRP1^fl/fl^ mice (LRP1-KO) or Nestin-Cre^ER^:td-tomato:LRP1^+/+^ (Control). Mice were subjected to MCAO at 3 months of age, 1-month after tamoxifen. IHC was performed in **(C)** control and **(D)** LRP1 KO mice, showing signal of td-tomato (red) colocalizing with LRP1 signal (green), together with orthogonal views. **(E)** LRP1 immunolabeling was quantified in td-tomato positive NSCs (*n* = 4-5 mice). **(F)** Cortical blood flow was measured in control (Cntrl, white) or LRP1-KO (gray boxes) (*n* = 18-20) and expressed as a percentage of pre-occluded blood flow. **(G)** mNSS at 6 h and 3 days post-stroke in *n* = 17-34 mice/group. Results are averages ± SEM. Significant differences were tested using ANOVA followed by Tukey’s HSD.

We used middle cerebral artery occlusion (MCAO) to create a transient (30-min), hemispheric ischemic stroke via 75% reduction in cortical blood flow. Blood flow returned to normal levels after removing the occlusion (Fig. 1A). We subjected mice to tamoxifen injection to induce Cre activation (Fig. 1B) in Control (Nestin-^CreERt2^:tdTomato:LRP1^+/+^) (Fig. 1C) and LRP1-KO (Nestin-Cre^ERt2^:tdTomato:LRP1^fl/fl^) (Fig. 1D) mice which resulted in near complete loss of LRP1 in NSCs at time of MCAO, 1 month later (*P* < .0001, Fig. 1E). The modified neurological severity score (mNSS) was used to test for functional impairment at 6 h and 3 days post-stroke. Regardless of genotype, mice exhibited similar cortical blood flow reductions (Fig. 1F) and mNSS score (Fig. 1G), suggesting that lesions were of similar severity in the acute period post-stroke.

**Figure 2.**
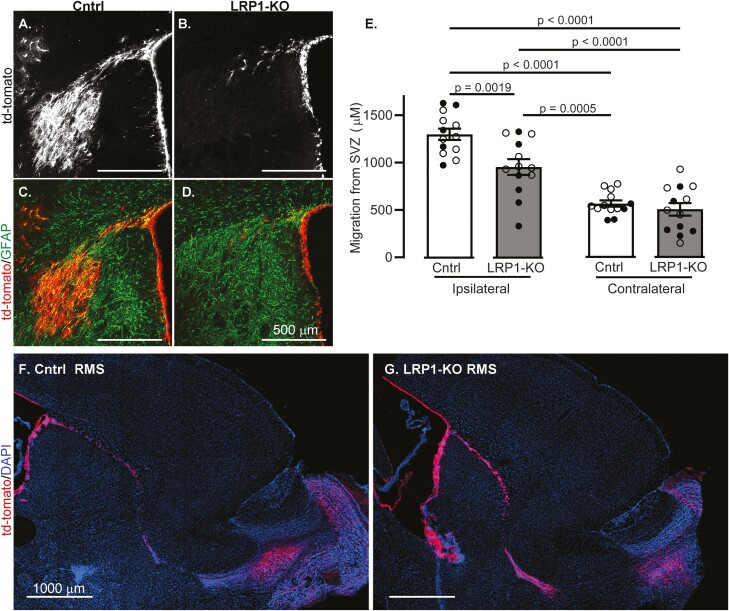
LRP1-KO in adult-born neuroblasts causes migration defects after ischemia. Coronal sections show representative image of tdTomato+ cells (white) 2 weeks after MCAO in **(A)** control and **(B)** LRP1 KO mice. TdTomato images (red) were merged with GFAP-labelled imaged (green) to show post-stroke glial scarring from **(C)** control and **(D)** LRP1-KO mice. **(E)** Quantification of tdTomato+ cell distance from the SVZ in ipsilateral and contralateral regions (*n* = 13). Results are averages ± SEM. Represented significant differences were tested using ANOVA followed by Tukey’s HSD. Representative images of the RMS in sagittal sections of **(F)** control and **(G)** LRP1-KO mice.

Mice were harvested 2 weeks post-MCAO, and the distance of tdTomato positive cells that traveled from the SVZ toward the striatal lesion was measured. NSCs lacking LRP1 had reduced localization toward the ischemic lesion compared to controls (Fig. 2A–2E, *P* < .0001). A depletion of the stem cell niche, or lack of proliferation could result in lower numbers of migrating tdTomato positive cells. To test this, we measured proliferation via EdU incorporation in non-stroked control (Supplementary Fig. S1A) and LRP1 KO (Supplementary Fig. S1B) mice and found that LRP1 KO mice had significantly more proliferating cells within the SVZ niche compared to control mice (*P* < .001, Supplementary Fig. S1C), and similarly had more tdTomato positive cells layered along the SVZ (*P* = .03, Supplementary Fig. S1D). To ascertain if the migration defect was due to a general inability to migrate, we examined if tdTomato positive cells were found in the olfactory bulb of non-stroked mice. Regardless of genotype, tdTomato positive cells could be found along the RMS and in the olfactory bulb (Fig. 2F, 2G).

### LRP1 Knock-Out Causes Loss of CXCR4

**Figure 3.**
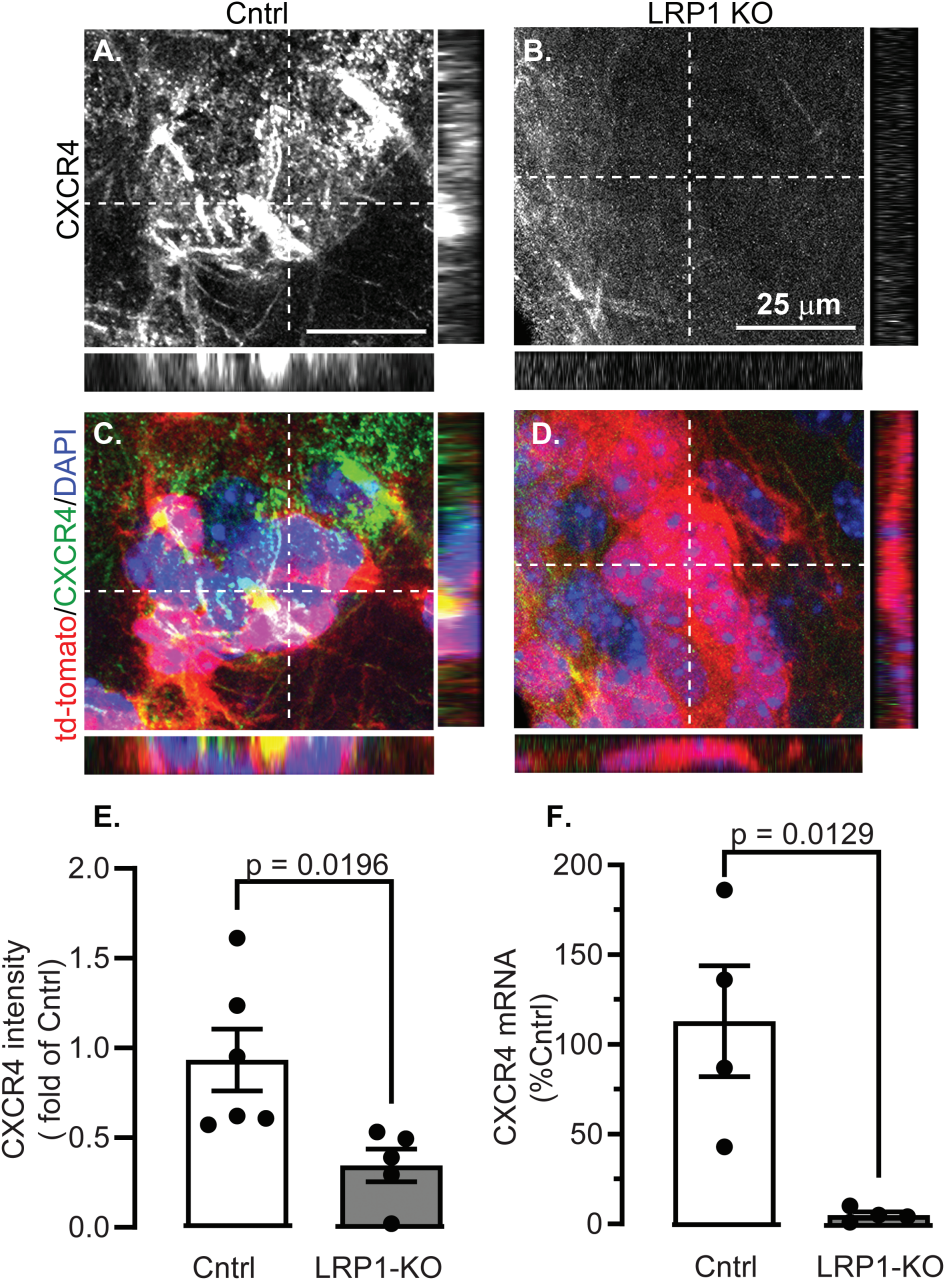
LRP1 KO decreases CXCR4 expression in adult NSCs. Representative images with orthogonal views are shown from SVZs in **(A, C)** control (Cntrl) and **(B, D)** LRP1-KO mice 2-weeks after MCAO of **(A, B)** CXCR4 immunostained cells, **(C, D)** channels were merged to show tdTomato (red), CXCR4 (green), and nuclear DAPI (blue). **(E)** Quantification of CXCR4 intensity in tdTomato+ cells (*n* = 5-6 mice/group). **(F)** qRT-PCR of CXCR4 was in tdTomato+ sorted cells (*n* = 4). Results are averages ± SEM, differences were tested using ANOVA followed by Tukey’s HSD.

We tested if CXCR4 expression was affected by LRP1-KO, as this pathway is necessary for neuroblast migration to ischemic lesions,^5^ but is less critical for directed migration along the RMS.^12^ We tested expression of CXCR4 and found only 30% of CXCR4 labeling in the red tdTomato positive cells in LRP1KO compared to control mice (*P* < .02, Fig. 3A–3E). We also measured CXCR4 in the NSCs of non-stroked mice and found mRNA expression reduced to approximately 5% of control in LRP1KO cells mice (*P* = .013, Fig. 3F). Finally, we induced LRP1KO in vitro by treating with cell-permeant Cre recombinase (Supplementary Fig. S2), and similarly found reduced CXCR4 immunolabeling (*P* < .05, Supplementary Fig. S3).

### LRP1 Knock-Out Impairs Migration to SDF1 In Vitro

**Figure 4.**
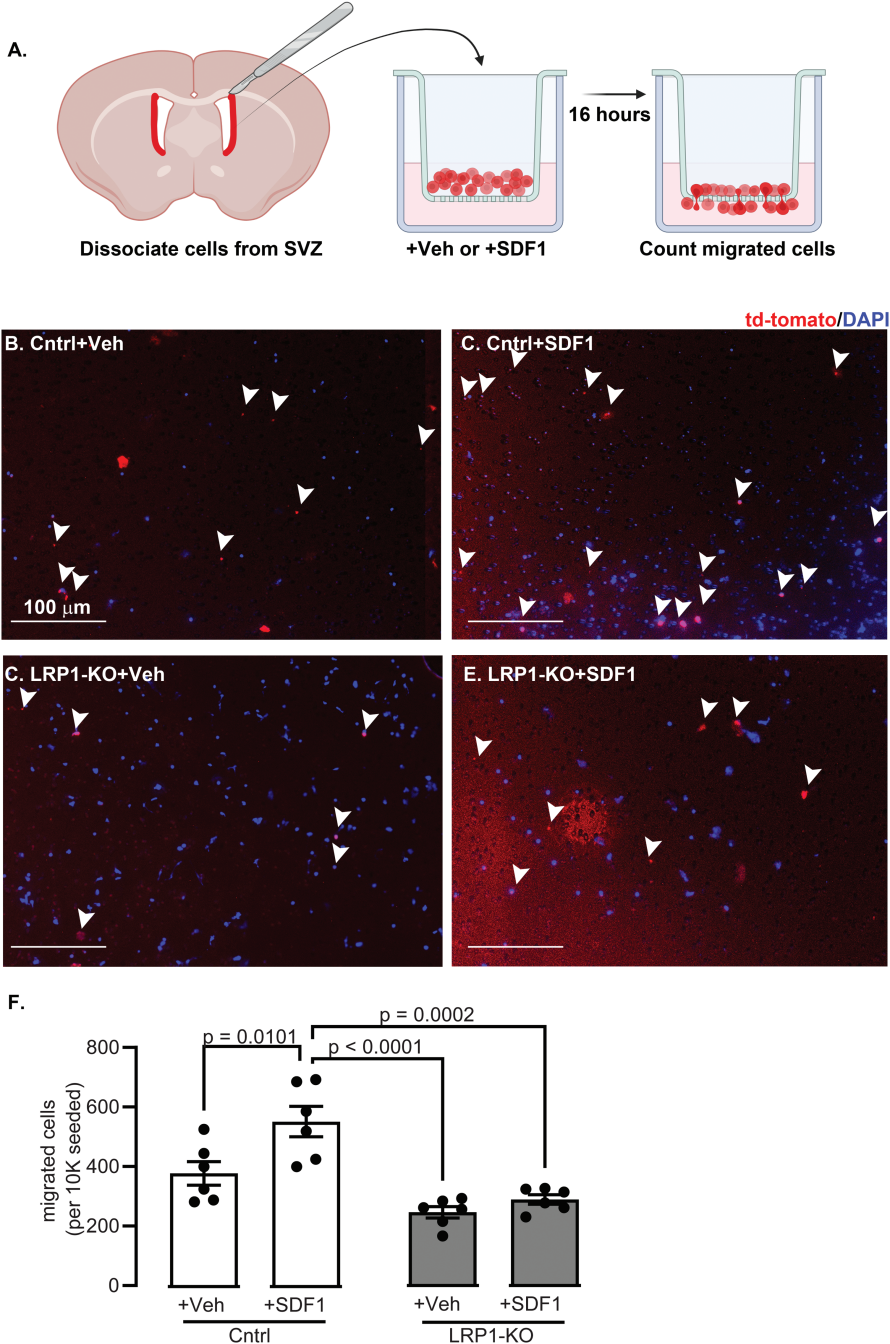
LRP1-KO prevents NSC migratory response to SDF1 in vitro. (**A**) Biorender.com-generated schematic of approach for chemotaxis assay with representative images for **(B)** control cells treated with vehicle **(C)** control cells with SDF1 **(D)** LRP1 KO cells with vehicle and **(E)** LRP1 KO cells with SDF1. White arrows show counted migrated cells **(F)** Quantification migrated cells (*n* = 6 wells/group, triplicate independent experiments). Results are averages ± SEM. Significant differences were tested using ANOVA followed by Tukey’s HSD and are represented on the graph.

The decreased localization of NSCs to the ischemic lesion suggests migration deficits in response to SDF-1 near lesions. To test if LRP1-KO impairs in vitro migration, NSCs were cultured from control and LRP1-KO mice and subject to a transwell migration assay (Fig 4A). In the presence of SDF1, significantly greater numbers of tdTomato positive control NSCs migrated through the chamber (*P* = .01). In contrast, SDF1 did not increase numbers of migrated tdTomato positive LRP1-KO NSCs (Fig 4B–4F).

## Discussion

Currently, little is known regarding a role of LRP1 in NSCs after ischemic damage. We discovered that loss of LRP1 in adult NSCs causes deficits in ischemia-induced migration, which was confirmed in vitro. While impaired proliferation could account for deficits in migration, we found that LRP1KO induced robust proliferation of cells in the SVZ, thus suggesting that proliferation cannot account for the observed migration deficits. In this study, we did not measure if in vivo differentiation after LRP1KO could account for migration deficits, but others have found altered in vitro differentiation after loss of LRP1,^13^ thus we cannot explicitly rule out this possibility. Nevertheless, we hypothesize that the observed reduction CXCR4 expression is the main factor that contributes to migration deficits.

LRP1 regulates many signaling pathways via interaction with many proteins.^8^ To our knowledge, our data is the first to implicate LRP1 in regulating CXCR4. Given the decrease in CXCR4 at both the mRNA and protein levels and a known role for LRP1 as a co-transcriptional activator,^9,14^ we hypothesize the regulatory mechanism to be transcriptional. Canonically, NRF-1 is the major positive transcriptional regulator of CXCR4 while Ying Yang 1 is the negative transcriptional regulator, but other transcription factors also alter CXCR4 expression.^15^ PPARγ downregulates CXCR4 in cancer-associated fibroblasts.^16^ This is of particular interest because LRP1 can be cleaved by γ-secretase, translocate to the nucleus, and act as co-activator for PPARγ.^14^ Thus, it is possible that PPARγ is intermediary in regulation of CXCR4 by LRP1. Future study is warranted. LRP1 could also regulate CXCR4 through plasma membrane receptor trafficking, which is a known method of CXCR4 regulation,^17^ thus LRP1 may also modulate this activity.

Our observations could have significant implications for tissue recovery after stroke. This report does not include such an analysis, however, interrogation into any differences in recovery is underway. Prior reports suggest that blocking SDF1/CXCR4 diminishes stem cell migration to lesions^5^ although these studies do not include analysis of the lesions. Other studies suggest that experimental elimination of NSCs is harmful to stroke recovery^,7^, thus homing to a lesion could be central to NSCs benefit. Future studies will help test this.

Aside from the benefits regarding our understanding of post-stroke migration to lesions, the implications of LRP1 regulation of CXCR4 are broad, as CXCR4 plays a vital role in multiple neurogenic and stem cell processes. For instance, CXCR4 plays an important role in hippocampal dentate gyrus precursor cell migration into the granule cell layer, both in development and also in adults.^18^ CXCR4 similarly enables stem cells to be retained within their respective niches, such as within the subventricular zone for NSCs^19^ or the bone marrow niche for hematopoietic stem cells.^20^ CXCR4 similarly plays a role in enabling proliferation and metastasis of some cancers^21^ Altogether, this novel regulation of CXCR4 by LRP1 could be manipulated to enhance our understanding of basic biological processes and similarly disease pathogenesis.

## Conclusion

Despite the study’s limitations, the implications of our discovery in the context of stroke recovery and basic neurobiology are significant. An intricate understanding of mechanisms underlying NSC response during ischemic injury may enable the development of therapeutics that improve natural neurogenic response to mitigate damage and promote recovery. Our discovery could also culminate in advancements in our collective understanding of basic NSC biology. Given that LRP1 knockout is embryonic lethal,^22^ it must play a fundamental role in regulation of NSCs, however, our understanding is somewhat limited. Safina et al reported the importance of LRP1 in neural stem cells in vitro*—*LRP1 deletion had a negative effect on survival and proliferation and altered differentiation to favor astrocytes and oligodendrocyte progenitor cells.^13^ Our studies extend upon such findings and suggest a multi-factorial role of LRP1 on NSC biology in vivo. Improved understanding of LRP1 in NSC biology will enhance our ability to acutely harness and regulate neurogenic processes, in turn broadly impacting a number of neurobiological diseases affected by neurogenesis, including schizophrenia,^23^ mood disorders,^23^ neurodegenerative diseases,^24^ and epilepsy.^25^

## Supplementary Material

sxad034_suppl_Supplementary_Material

## Data Availability

The data underlying this article will be shared on reasonable request to the corresponding author.
