# Supplementary material for: Loss of LRP1 in Adult Neural Stem Cells Impairs Migration to Ischemic Lesions
Source: Stem Cells. 2023 Apr 25;41(6):570–7. doi: 10.1093/stmcls/sxad034 (PMC10267954; doi:10.1093/stmcls/sxad034)
Supplement: sxad034_suppl_Supplementary_Material [file sxad034_suppl_supplementary_material.pdf]

Supplemental Information:

Neural Stem Cell Culture:

Lateral walls of the lateral ventricle were microdissected from 2-4 month old male and female mice and dissociated with 0.25% papain and 12 µg/mL DNase at 37 degrees C for 45 minutes followed by manual titration. Cells were washed with DMEM 3 times and plated on poly-L-ornithine coated culture dishes. Cells were cultured in serum-free DMEM with additives (1 mM L-glutamine, sodium pyruvate, B-27 (Gibco 17504-044), N2 (Gibco 17502-048), N-acetyl-cysteine) supplemented with basic fibroblast growth factor (FGF2; Peprotech, 10 ng/mL 450-33). Neural stem cells were maintained by re-feeding every 2-3 days with fresh media containing FGF2.

Chemotaxis Assay

Acutely dissociated SVZ neural stem cells were collected and processed for cell culture similar to previously published protocols[1]. A total of 21,600 cells were plated onto the upper wells (n=6/condition) of 96-well poly-l-ornithine (PLO) coated chemotaxis chambers (10 µm pore size, Neuro Probe Inc) and maintained in DMEM containing l-glutamine (1 mM), sodium pyruvate (1 mM), B-27 (1X from Gibco), N2 (1X from Gibco). The bottom chamber of the wells was loaded with either serum free media or serum free media with SDF1 (500nM). Cells were incubated overnight (37°C, 5% CO2), and then the migrated tdTomato positive cells were counted. Recombination rate was determined by counting the ratio of tdTomato positive/DAPI positive cells from NSCs cultured in-tandem on PLO coated Terasaki plates. Migrated cells were normalized using the recombination rate. Results are pooled from triplicate independent experiments and were analyzed by a researcher blinded to experimental genotype.

In vitro knockout of LRP1 and immunocytochemistry

For experiments involving *in vitro* knock-out of LRP1 and immunocytochemistry, isolated cells were plated onto poly-L-ornithine coated glass coverslips (~50,000 cells/coverslip) and were refed at 48 hours post-isolation with recombinant HTN-Cre (Excellgen; 0.5µM) for 24 hours. Neural stem cells were grown for 14 days in culture prior to harvest. At harvest, cells were washed in PBS 3 times, fixed with 4% paraformaldehyde in PBS for 5 minutes, permeabilized with 0.25% triton in PBS, then washed in PBS. Non-specific binding was blocked with 5% bovine serum albumin in PBS, and primary antibodies were incubated overnight in blocking buffer at 4 degrees C. Cells were washed again (3X10min with PBS), and incubated with species-specific secondary antibody in blocking buffer. Cells were washed in PBS, and nuclei were stained with DAPI (5mg/ml) prior to mounting on glass slides with Aquapolymount for imaging on a Zeiss LSM 710 laser scanning microscope. Z-stacks were taken of at least 50 cells/well (n=3-7 wells per experiment), and intensity of immunolabeling was determined in vehicle-treated or HTN-Cre-treated tomato-positive cells by measuring the gray values and percentage of pixels above background threshold in individual cells. Results for each well were pooled and expressed at a % of vehicle-treated values.

Proliferation measurements

Two-hours prior to harvest, 3 month old mice were injected with 50mg/ml EdU (Sigma 900584 10mg/ml in sterile saline), and mice were harvested for immunohistochemistry as described. Proliferating cells were labelled using Click-it EdU Imaging Kit (Invitrogen C10338) according to manufacturer's instructions, and then lateral ventricles were imaged on a Zeiss LSM 710 laser scanning microscope. Images were analyzed by a blinded researcher who counted the number of EdU positive proliferating td-tomato positive cells and EdU negative td-tomato positive cells in the lateral ventricles from 10-13 slices per mouse (n=3 mice) and were expressed as a ratio of EdU positive/ td-tomato positive cells.

In vivo Immunohistochemistry

Following intracardial perfusion with PBS followed by 4% PFA, brains were removed and incubated overnight in 4%PFA, brains were then placed in 30% sucrose/PBS for at least 3 days. Tissue was immersed in OCT (Optimal Cryo-Temp, Tissue Plus – Fisher HealthCare) and frozen using isobutane surrounded by liquid nitrogen. Coronal slices (30 µm) were mounted to gelatin coated slides, dried overnight then rehydrated in PBS prior to permeabilization in 0.2% triton in PBS for. Slides were washed with PBS. Autofluorescence was blocked with Sudan Black (0.1% in 70% EtOH, 5 min) and washed with PBS until clear. Non-specific protein binding was blocked with 5% BSA for 45 min). Primary antibodies were diluted in blocking solution, and tissues were incubated overnight at 4ºC. Slides were washed with PBS before incubation in species-specific secondary antibodies in blocking solution (1 hour RT). Slides were washed in PBS (2X5 min), stained with DAPI (5mg/ml for 5 min, washed with PBS and then rinsed in distilled water. Slides were dried and mounted to coverslips with Aqua-Poly/Mount (18606-20).

Antibodies used were rat anti-CXCR4 (R&D MAB21651 1:25); chicken anti-GFAP (Sigma AB5541 1:500); rabbit anti-LRP1 (Abcam ab92544), donkey anti-chicken Alexa 488 (Jackson 703-545-155 1:200); donkey anti-rat Alexa 488 (Jackson 711-545-152 1:200).

RNA extraction and qPCR

Similar to previously published protocols[1], the SVZ's (n=4) were micro-dissected and suspended in ice cold hibernation buffer [30mM potassium chloride (5mM sodium hydroxide (5mM sodium phosphate monobasic monohydrate, 0.5mM magnesium chloride hexahydrate, 20mM sodium pyruvate, 5.5mM d+ dextrose anhydrous and 200mM d-sorbitol and dissociated with 10 units of papain and 10µL of DNase in DMEM. Cells were triturated, washed, and pelleted then resuspended in FACS Buffer (30% Glucose, 10% BSA in HBSS) and sorted into RNA Later (Invitrogen AM7020) using BD FACSAria to select only tdTomato+ cells. RNA was harvested using TRIzol (Ambion 15596018). First-strand synthesis was achieved using the Applied Biosystem High-Capacity cDNA reverse transcription kit according to manufacturer's instructions. QPCR was run in triplicate reactions using iTaq SYBR Green Supermix (BioRad 172-5120) according to manufacturer's instructions on Bio-Rad CFX96 Touch Real-Time PCR detection system. Relative levels of mRNA were determined after normalizing loading via the delta-delta Ct method.

**Primers**	**Forward**	**Reverse**
GAPDH	AGGTCGGTGTGAACGGATTTG	TGTAGACCATGTAGTTGAGGTCA
CXCR4	AGCAGGTAGCAGTGAAACCTCTGA	TGGTGGGCAGGAAGATCCTATTGA

Image analysis

Images were obtained using a Zeiss LSM710 laser scanning confocal microscope or Zeiss Axio Observer.D1. Image analysis was performed by a researcher blinded to genotype or experimental manipulation using ImageJ containing the Fiji plug-ins suite.

To measure CXCR4 expression intensity, tdTomato images were used to create region of interest (ROI) selections to limit analysis to Cre+ NSCs: 8-bit images were subject to contrast enhancement to visualize cells, despeckle was used (2X) to remove single pixels, and then was thresholded to include only red signal. The "analyze particles" function created cell masks (>20 pixels). Masks were dilated (2X) and then used to create a selection to add to the ROI manager. ROIs from the tdTomato channel were used to select the same area in the 8-bit CXCR4-positive

channel, and the “measure” function measured gray values. For each mouse, z-stacks from at least 3 separate images were measured, and the results are pooled from n=5-6 individual mice.

For *in vivo* migration, the distance between the SVZ and the furthest migrating neuroblast in the coronal plane was measured from the edge of the dorsolateral corner of the SVZ along the center of the migratory track cells to the furthest tdTomato positive cell. Results are pooled from of 3-5 slices/animal of n=13 mice/group.

Supplemental Figures:

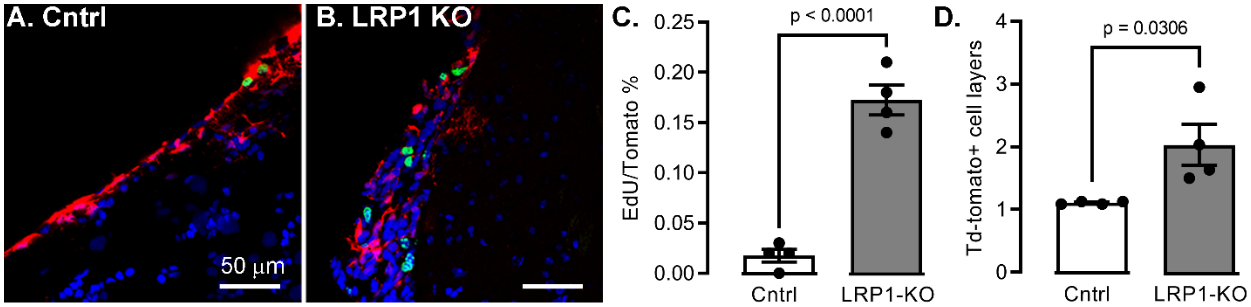

Figure S1: LRP1 KO causes elevated proliferation in the subventricular zone of non-stroked mice. Coronal sections show representative image of tdTomato+ (red), EdU+ (green) cells and DAPI (blue) nuclear staining in SVZ of **(A)** Control (Cntrl) and **(B)** LRP1 KO mice one-month after tamoxifen injection. **(C)** Percent of Td-tomato+ cells that are dual-labeled for EdU. **(D)** Total cross-sectional layers of Td-tomato+ cells arrayed next to the lateral ventricles. Results are averages ± SEM from 10-13 images/mouse, n=4 mice/group. Significant differences were tested using student's T-test.

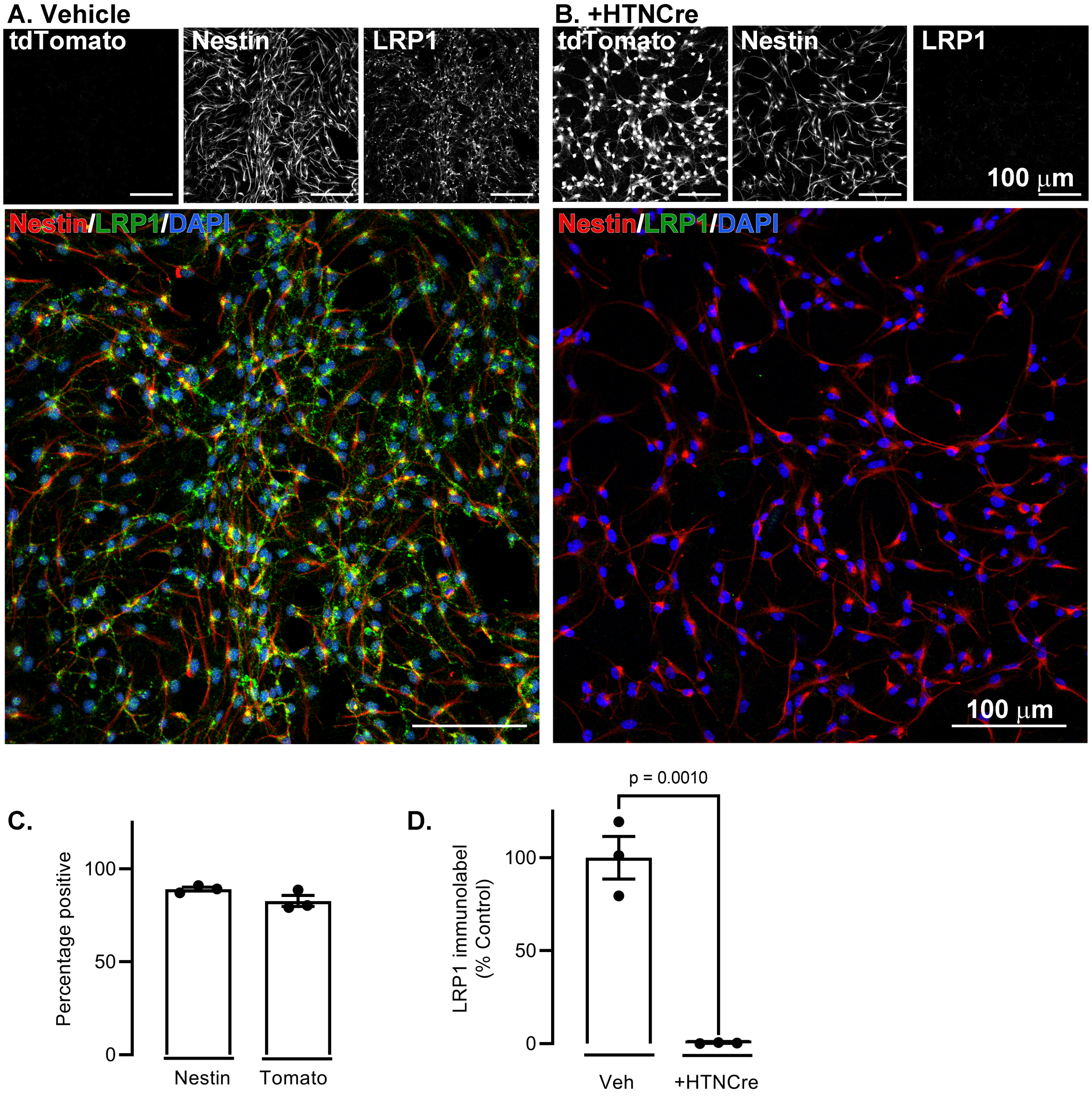

Figure S2: **Immunolabeling of LRP1 in NSCs treated with recombinant cell-permeant Cre *in vitro*.** Representative images from cultured NSCs that were **(A)** vehicle-treated or **(B)** HTNCre-treated show td-tomato (upper left, red), Nestin (upper middle), LRP1 (upper right, green) and DAPI nuclear staining (blue). **(C)** Quantification of the percentage of Nestin positive and td-Tomato positive cells and **(D)** amount of LRP1 immunolabeling represented as a percentage of control (n=3 coverslips/group). Results are averages ± SEM. Differences were tested using student's T-test.

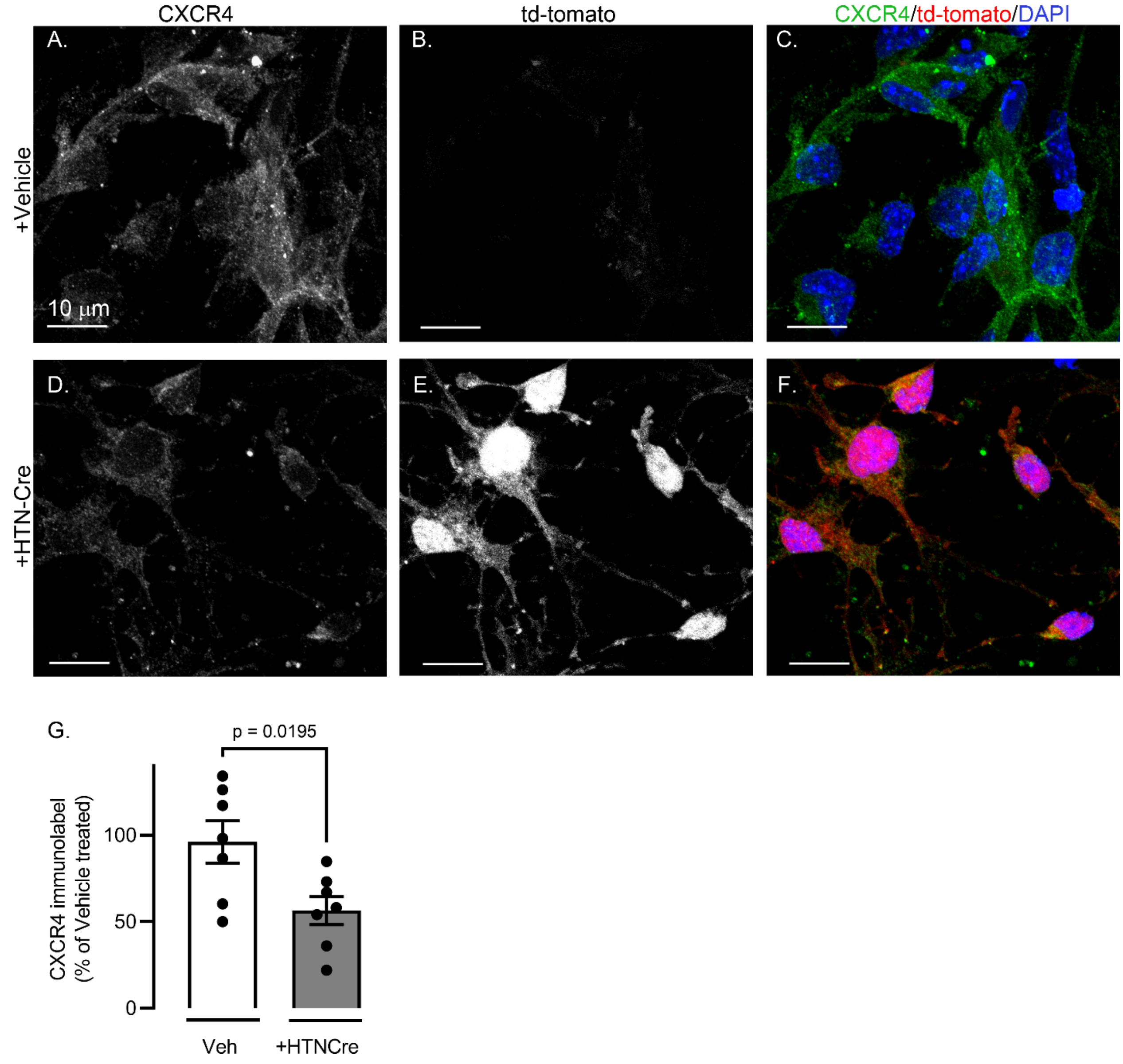

Figure S3: **LRP1 KO results in loss of CXCR4 immunolabeling *in vitro* after treatment with recombinant cell-permeant Cre.** Representative images from cultured NSCs that were **(A-C)** vehicle-treated or **(D-F)** HTNCre-treated show **(A,D)** CXCR4 immunolabeling **(B,E)** td-tomato expression, or **(C,F)** merged image of CXCR4 (green), td-tomato (red), and nuclear DAPI (blue). **(G)** Quantification of CXCR4 immunolabeling represented as percentage of control (n=7 coverslips). Results are averages ± SEM. Differences were tested using student's T-test.

Supplemental Citation:

1 Kokovay, E. *et al.* Adult SVZ lineage cells home to and leave the vascular niche via differential responses to SDF1/CXCR4 signaling. *Cell stem cell* **7**, 163-173, doi:10.1016/j.stem.2010.05.019 (2010).
